# Establishment of an Indirect Competitive Enzyme-Linked Immunosorbent Method for the Detection of Heavy Metal Cadmium in Food Packaging Materials

**DOI:** 10.3390/foods10020413

**Published:** 2021-02-13

**Authors:** Xianshu Fu, Erjing Chen, Biao Ma, Ying Xu, Peiying Hao, Mingzhou Zhang, Zihong Ye, Xiaoping Yu, Chaofeng Li, Qingge Ji

**Affiliations:** Zhejiang Provincial Key Laboratory of Biometrology and Inspection & Quarantine, College of Life Science, China Jiliang University, Hangzhou 310018, China; fxs@cjlu.edu.cn (X.F.); s1709071002@cjlu.edu.cn (E.C.); mb@cjlu.edu.cn (B.M.); clsxuying@163.com (Y.X.); haopy@cjly.edu.cn (P.H.); zhye@cjlu.edu.cn (Z.Y.); yxp@cjlu.edu.cn (X.Y.); lcf609162059@163.com (C.L.); QinggeJi1997@163.com (Q.J.)

**Keywords:** food packaging materials, cadmium (Cd), monoclonal antibody, ic-ELISA, ICP-AES, rapid detection

## Abstract

Heavy metals in food packaging materials have been indicated to release into the environment at slow rates. Heavy metal contamination, especially that of cadmium (Cd), is widely acknowledged as a global environment threat that leads to continuous growing pollution levels in the environment. Traditionally, the detection of the concentration of Cd relies on expensive precision instruments, such as inductively coupled plasma mass spectrometry (ICP-MS) and inductively coupled plasma-atomic emission spectrometry (ICP-AES). In this study, an indirect competitive enzyme-linked immunosorbent assay (ic-ELISA) based on a specific monoclonal antibody was proposed to rapidly detect Cd. The half-inhibitory concentration and detection sensitivity of the anti-cadmium monoclonal antibody of the ic-ELISA were 5.53 ng mL^−1^ and 0.35 ng mL^−1^, respectively. The anti-Cd monoclonal antibody possessed high specificity while diagnosising other heavy metal ions, including Al (III), Ca (II), Cu (II), Fe (III), Hg (II), Mg (II), Mn (II), Pb (II), Zn (II), Cr (III) and Ni (II). The average recovery rates of Cd ranged from 89.03–95.81% in the spiked samples of packing materials, with intra- and inter-board variation coefficients of 7.20% and 6.74%, respectively. The ic-ELISA for Cd detection was applied on 72 food packaging samples that consisted of three material categories—ceramic, glass and paper. Comparison of the detection results with ICP-AES verified the accuracy of the ic-ELISA. The correlation coefficient between the ic-ELISA and the ICP-AES methods was 0.9634, demonstrating that the proposed ic-ELISA approach could be a useful and effective tool for the rapid detection of Cd in food packaging materials.

## 1. Introduction

Due to its favourable toughness and ductility [1,2,3], cadmium (Cd) is becoming one of the most frequently used elements in the manufacturing of metal, plastic and dyestuff materials, which are commonly used in food packaging, kitchenware, plastics, bottles and boxes [4,5]. Increased public awareness of contamination in food packaging materials has raised global concerns regarding potential heavy metal environmental contamination from packaging [6,7]. Cd pollution has now been identified as one of the most major factors affecting food safety in many countries [8,9,10], especially in nori (restaurant-served and ready-to-eat sushi in the Polish market) [11]. Perhaps more worrisome, massive over-packaging has led to the growing presence of toxic metals such as cadmium, mercury, and lead [12], which now present extreme or even incredible toxicity. Therefore, the quality and safety of food packing materials are closely related to people’s health and happiness [13]. To strengthen the treatment and management of cadmium pollution, it is imperative to regulate the arbitrary discharge of cadmium materials [14].

Cd in the environment can enter and accumulate in humans’ bodies through food, water and air [15]. When Cd enters the body, it is transported into the bloodstream via erythrocytes and albumin, and is then accumulated in the kidneys [16], lungs [17], and gut [18]. The amount of heavy metals that humans can bear daily is very limited [19], and the presence of these heavy metals in the human body is a severe problem even at low or trace concentration levels [20]. Exceeding this threshold can destroy the kidneys, bones, heart and blood vessels of the human body, causing adverse reactions, such as carcinogenesis [17], coronary heart disease [21], cardiovascular disease [22], mutagenesis and teratogenesis [23].

In 1973, the Food and Agriculture Organization of the United Nations (FAO) and the World Health Organization (WHO) listed Cd as a mandatory monitoring index at the Food Additives and Pollutants Conference. Cadmium also was included as a key monitoring element by China’s food safety agency. According to China National Standards (GB/T 31604.24-2016, National standard for food contact materials and products) [24], the detection limit and quantitation limit are 30 mg kg^−1^ and 100 mg kg^−1^, respectively, for graphite furnace atomic absorption spectrometry (GFAAS), and 7 mg kg^−1^ and 20 mg kg^−1^, respectively, for inductively coupled plasma mass spectrometry (ICP-MS). At the end of 2019, European Union (EU) has promulgated new regulations (Restriction of Hazardous Substances (RoHS)) for limiting cadmium pollution. The EU limits the detection limit of Cd concentration (≤100 mg kg^−1^).

Traditionally, inductively coupled plasma mass spectrometry (ICP-MS) [25], inductively coupled plasma-atomic emission spectrometry (ICP-AES) [26], room temperature ionic liquids (RTILs), and speciated isotope dilution mass spectrometry (SID-MS) [27,28] are frequently applied for trace cadmium ion detection. These detection methods characteristically have high accuracy, specificity and sensitivity. Nevertheless, the high price of the above-mentioned detection devices limits their actual applicable scenarios. Meanwhile, these approaches are not suitable for on-site spot checks of market products, and they also fail to meet the requirements for rapid customs clearance of products [29]. Therefore, it is imperative to develop and establish a convenient and rapid trace cadmium detection method [30,31].

In recent years, the immunological detection method of heavy metals has been demonstrated to be more suitable for large-scale sample detection, such as in a Cd detection kit, due to its advantages of rapid detection speed, low cost and a good application prospect [32,33]. Over the past decade or so, immunoassays have been widely applied in various methods to detect of various heavy metals [34], such as immunochromatographic kits [35], fluorescent-labeled immunoassays [36,37,38] and nanoparticle-labeled immunoassays [39,40]. However, due to the complex sample preparation process, the above detection methods are often limited to the research of detection methods and the application of water sample detection [41], which has influenced the development of the rapid detection of heavy metals in food packaging materials.

In this paper, an ic-ELISA based on anti-Cd monoclonal antibody is presented to detect Cd in food packing materials. The results show that this method is in good agreement with ICP-AES in food packing materials samples and has an extremely high practical application value.

## 2. Materials and Methods

### 2.1. Reagents, Chemicals and Samples

Al (III), Ca (II), Cu (II), Fe (III), Hg (II), Mg (II), Mn (II), Pb (II), Zn (II), Cr (III), Ni (II) and Cd (II) (1 mg mL^−1^ concentration of the above reagents) standard products were purchased from the National Institute of Metrology, P. R. China (Beijing, China). Ovalbumin (OVA), bovine serum albumin (BSA), Tween-20, Goat anti-rat–mouse HRP, Dimethyl sulfoxide (DMSO), Freund’s complete and incomplete adjuvants (cFA and iFA) and 4-(2-hydroxyethyl)piperazine-1-ethanesulfonic acid N-(2-hydroxyethyl)piperazine-N’-(2-ethanesulfonic acid) (HEPES) were bought from Sigma-Aldrich (St. Louis, MO, USA). 3,3′,5,5′-Tetramethylbenzidine (TMB) was gained from Dean Biological Co., Ltd. (Zhejiang, China). RPMI 1640 cell culture medium, fetal bovine serum (FBS), HAT supplement (liquid mixture of sodium hypoxanthine, methotrexate, and thymine) and HT supplement (liquid mixture of sodium hypoxanthine and thymidine) were obtained from Life Technologies Co. (New York, NY, USA). 1-(4-isothiocyanatobenzyl) vinyldiamine-N,N,N’,N’-tetraacetic acid (ITCBE) was acquired from Dongren Chemical Technology Co., Ltd. (Shanghai, China). The myeloma cell line of Sp2/0 was obtained from the Chinese Academy of Sciences (Shanghai, China). Cell culture plates and 96-well micro-titer plates were obtained from Corning Inc. (New York, NY, USA). We paid Sinopharm Group Chemical Reagent Co., Ltd. (Shanghai, China) for other conventional chemical reagents.

A phosphate buffer saline (PBS buffer, 10 mM, pH 7.4) was prepared by dissolving 0.2 g NaCl, 1.55 g NaH_2_PO_4_ and 0.25 g KH_2_PO_4_ in ultra-pure water and diluted to 1 L. Phosphate buffer solution (PBST) was obtained from a PBS buffer (10 mM, pH 7.4) containing 0.05% Tween-20. HEPES (2.649 g) and ITCBE (4.136 g) were dissolved in ultra-pure water and diluted to 1 L, resulting in a HEPES buffer of 10 mM, with pH 9.0. The solution is adjusted to pH 9.0 using an NaOH solution. The food packing material samples were either presented by Professor Jun Wang (Jiangnan University, Wuxi, China) or bought from a supermarket in Hangzhou (China).

72 food packaging materials were collected for Cd detection. These 72 samples could be classified into three categories: ceramic, glass and paper. For each category, 24 samples were prepared. The ceramic group included six ceramic water cups, six ceramic bowls, six ceramic pots and six flat ceramic basins. The glass group included six glass water cups, six glass jars, six small glass wine cups and six high foot glass wine cups. The paper group included six paper trays, six paper cups, six paper bowls and six paper plates. All 72 samples were purchased from the supermarket.

### 2.2. Preparation of Cd-ITCBE-BSA and Cd-ITCBE-OVA

**Figure 1:**
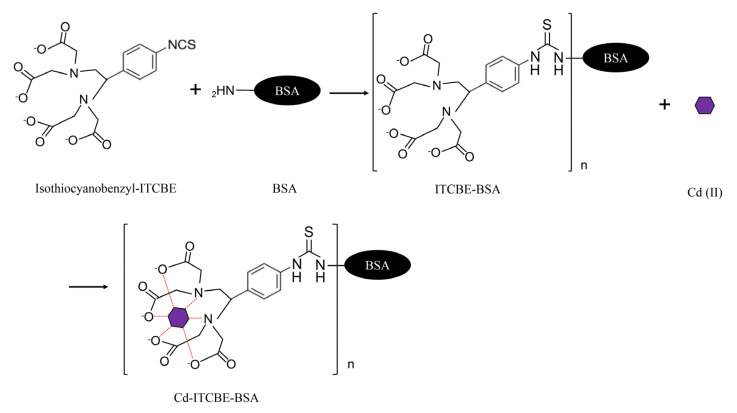
Synthesis scheme of Cd (II) artificial antigens.

Complete antigens were reacted according to Kong et al. [42] and modified with a bi-functional chelating agent ITCBE. The comprehensive synthesis scheme is shown in Figure 1. Cd-1-(4-isothiocyanatobenzyl) vinyldiamine-N,N,N’,N’-tetraacetic acid (ITCBE)-ovalbumin(OVA) (Cd-ITCBE-OVA) and Cd-1-(4-isothiocyanatobenzyl) vinyldiamine-N,N,N’,N’-tetraacetic acid (ITCBE)-bovine serum albumin (BSA) (Cd-ITCBE-BSA) were used as an immunogen and a coating antigen, respectively. Briefly, 2.0 mg ITCBE was dissolved in 0.2 mL DMSO and mixed with 10.0 mg BSA in 10.0 mL HEPES buffer, and then subsequently stirred at room temperature for 24 h. At the end of the reaction, the samples were collected and dialyzed at 4 °C for 24 h with the HEPES buffer in order to purify. After dialysis, the Cd (II) standard solution (1.2 mL, 1 mg/mL) was added drop by drop and the pH was adjusted to 9.0 with NaOH, then stirred at room temperature for 4 h. The conjugates were dialyzed in a HEPES buffer at 4 °C for 24 h, and subsequently in a phosphate buffer (PBS buffer) at 4 °C for 24 h. Afterwards, the collected samples were stored at −20 °C at the dark and were ready to be used. Using OVA instead of BSA, CD-ITCBE-OVA was synthesized in the same way.

### 2.3. Production of Monoclonal Antibody

This study strictly complied with the Regulations of Zhejiang Province on the Management of Experimental Animals (2009) to minimize the suffering of animals. Antibodies were generated in Balb/c mice, as described by Gong et al. [43]. Three Balb/c female mice were purchased from the experimental Animal Centre of Hangzhou Normal University (Hangzhou, China), immunized with Cd-ITCBE-OVA (200 μg in 100 μL sterile PBS) by sub-cutaneous injection and emulsified with an equal volume of Freund’s complete adjuvant. In subsequent immunizations (three times, at an interval of every 2 weeks), 100 µg of Cd-ITCBE-OVA conjugate (in 100 µL sterile PBS) with the same volume of Freund’s incomplete adjuvant was used. Blood was collected from the posterior orbital vein and stored at 4 °C overnight. Afterwards, the anti-serum was extracted from the blood and centrifuged at 5000 rpm for 8 min. Anti-cadmium monoclonal antibodies were produced by the hybridoma technique. In an RPMI 1640 cell culture medium supplemented with 10% FBS, murine myeloma cells Sp2/0 were maintained at the exponential growth stage. Immune mouse spleen cells were fused with myeloma cells. Hybridomas were selected in a HAT medium (dulbecco’s modified eagle medium (DMEM) containing 15% FBS). The cultures of the 96-well plates were maintained in an incubator with 5% CO_2_, and the hybridoma colonies were subsequently expanded in the culture medium. The supernatant of the hybridoma was screened by ic-ELISA, with 10 ng mL^−1^ cadmium similarly screened for comparison. The established ELISA system is used to select specific cell lines against Cd, and sub-clones are used to continuously cultivate multi-cell line wells to specific single-cell line wells. Then, the stable cells were expanded and cryopreserved in liquid nitrogen. The stable cells were produced for ascites and purified via the octanoic acid–sulfur ammonium method to obtain monoclonal antibodies. The supernatant of hybridoma was screened by ic-ELISA, with 10 ng mL^−1^ cadmium similarly screened for comparison.

A Cd-ITCBE-BSA conjugate was used as the coating coupling. A continuous dilution method was introduced to sub-clone the hybridoma secreting cadmium specific antibody. Colonies of interest were propagated, frozen (kept overnight at −80 °C) in a culture medium containing 10% DMSO and preserved in liquid nitrogen.

The affinity of monoclonal antibodies was determined with ic-ELISA by referring to Kim et al. [44]. The affinity of the monoclonal Abs (K) was as Equation (1):

(1) K = C_Ab-Ag_/(C_Ab_*C_Ag_).

where C_Ab-Ag_, C_Ab_ and C_Ag_ are the concentrations of the conjugate, antibody and anti-gen, respectively.

### 2.4. Optimal Working Concentration of Anti-Cadmium Monoclonal Antibody and Coating Anti-Gen

96-well plates coated with Cd-ITCBE-BSA were blocked at 4 °C for 24 h [45]. After the reaction, the 96-well plates were dried at 37 °C for 5 h and stored at 4 °C for later use. PBS and the diluted Cd (II) standard material were added to seated 96-well plates at a concentration of 50 μL per well. Afterwards, the anti-cadmium monoclonal antibody and goat anti-mouse-horseradish peroxidase were diluted and added to the closed 96-well plates with 50 μL per well. The reaction took place at 25 °C for 30 min, and the plates were washed with PBST three times after the reaction was completed. The TMB chromogenic reagent was then added to the 96-well plates at a concentration of 100 μL per well. The reaction was performed at 25 °C for 10 min, and the stopping solution was added to the plate at 50 μL per well. The OD_450_ value was measured at 450 nm with a micro-plate reader [46]. The appropriate OD_450_ value was selected, and the optimal working concentration was determined based on the inhibition rate of the Cd (II) standard. 

### 2.5. Standard Curve and Sensitivity Analysis

The optimal working concentration of the anti-cadmium monoclonal antibody and goat anti-mouse-horseradish peroxidase were determined based on Liu’s reference [47]. The specific concentrations of Cd (II) standard substance (0, 0.33, 1.0, 3.0, 9.0, 27.0 and 81.0 ng mL^−1^) and goat anti-mouse-horseradish peroxidase were added to the blocked 96-well plates, respectively, and the reactions were carried out at 25 °C for 30 min. After the reaction, the 96-well plate was washed with PBST three times and the TMB chromogenic reagent was added to the plate at a concentration of 100 μL per well and subsequently performed at 25 °C for 10 min. The stop solution was added to the 96-well plate at 50 μL per well, and the OD value was measured at 450 nm with a micro-plate reader. The experiment was repeated 12 times. The logarithm of Cd (II) standard concentration and binding ratio were set as the abscissa and ordinate, respectively. The semi-logarithmic standard curve and regression standard curve were established, and the half-inhibition concentration was IC_50_. IC_50_ is a very important datum in the ic-ELISA standard curve, which is an S-shaped curve. In ic-ELISA, the OD value of the control well without an inhibition substance is B0, and that of the well with an inhibition substance is B. B/B0% is known as the binding rate, and the corresponding inhibitory substance concentration is called IC_50_ when the binding rate is 50%. The half-inhibitory concentration (IC_50_) and detection sensitivity (IC_10_) values of 50% and 10% inhibition rates were calculated by the formula, and the sensitivity of the standard curve was analysed.

### 2.6. Specificity Analysis of Ic-ELISA

Ic-ELISA was applied to detect an anti-cadmium monoclonal antibody and Cd (II), Al (III), Ca (II), Cu (II), Fe (III), Hg (II), Mg (II), Mn (II), Pb (II), Zn (II), Cr (III) and Ni (II), respectively. IC_50_ and cross-reaction rate (CR) were calculated. The antibody specificity was determined according to the cross-reactivity [48].

### 2.7. Minimum Detection Limit and Minimum Limit of Quantification

A suitable variety of food packing materials samples were selected from the market and tested by ICP-AES. From the results, 10 food packing materials samples without Cd (II) were randomly selected and identified as negative samples. Negative samples were examined 10 times using the ELISA method established in this study. The mean value of the negative ELISA samples assay plus three standard deviations (SD) was taken as the limit of detection (LOD). The mean value of negative ELISA sample test plus six times the standard deviation (SD) was represented as the minimum limit of quantitation (LOQ).

### 2.8. Precision and Accuracy of Ic-ELISA Analysis

The precision of the ic-ELISA could be reflected by the fluctuation of the OD_450_ value of each well in the plate and the OD_450_ value of the same concentration between the plates [42]. That is to say, the coefficient of variation (CV) was calculated by measuring the OD_450_ value, and each group of values came from 12 parallel experiments.

The accuracy analysis of ic-ELISA established in this study could be verified by adding the recovery rate method. Cd (II) standards were added to these negative food packing material samples at gradient concentrations of 0, 100, 200 and 400 ng mL^−1^, respectively. Then, the negative food packing material samples were detected via ICP-AES. The recovery rate was calculated for analysis [49]. Ten parallel experiments were respectively repeated to calculate the average value added and recovery rate after extraction. The ratio of actual sample concentration to theoretical concentration was used as the recovery rate.

### 2.9. Comparison of Ic-ELISA with ICP-AES

To verify the applicability of ic-ELISA, ICP-AES and ic-ELISA were used to analyse the spiked and actual samples of food packing material, respectively. 400 µL of 10% HCl and 400 µL of 0.1 M HNO_3_ were added to a 200 mg food packing material debris sample. The mixture was shaken well and placing it in a 100 °C constant temperature metal bath for 10 min. After heating, the supernatant was centrifuged at 13,000 rpm for 10 min. Soon afterwards, the same volume of the HEPES buffer was added to the neutralization solution for standby use.

The Cd (II) standard was added to the 10 negative samples in order to achieve the final specific concentrations (50, 100, 200 and 400 ng mL^−1^). These above added negative samples were analysed by ic-ELISA and ICP-AES, respectively.

ICP-AES and ic-ELISA were used to detect 12 kinds of food packing material samples. According to the test results, the test results of ic-ELISA and ICP-AES were used as the ordinate and abscissa, respectively. The regression curve was established with respect to the test results of the two methods. The correlation between the two methods was reflected by the correlation coefficient (R^2^) in the regression equation.

## 3. Results and Discussion

### 3.1. UV-Scanning Identification of Cd-ITCBE-BSA and Cd-ITCBE-OVA

**Figure 2:**
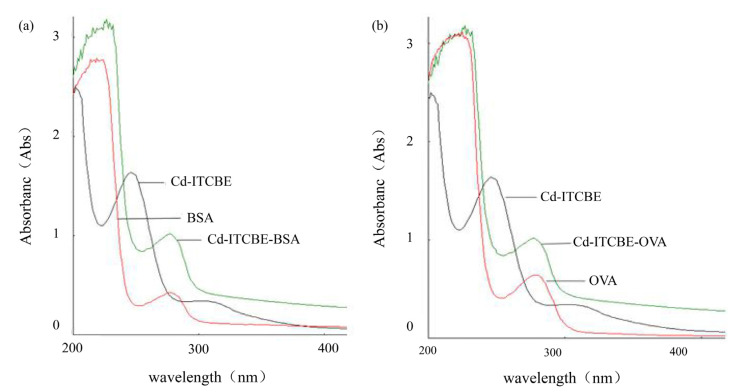
UV identification of Cd-1-(4-isothiocyanatobenzyl) vinyldiamine-N,N,N’,N’-tetraacetic acid (ITCBE)-bovine serum albumin (BSA) (**a**) and Cd-1-(4-isothiocyanatobenzyl) vinyldiamine-N,N,N’,N’-tetraacetic acid (ITCBE)-ovalbumin (OVA) (**b**) in the protein mixture.

The prepared Cd-ITCBE-BSA and Cd-ITCBE-OVA were identified using UV scanning [50]. The characteristic peak of Cd-ITCBE was found at 245.0 nm (1.43 mg/mL) and that of BSA was found at 280.0 nm (1.0 mg/mL). However, the coated anti-gen Cd-ITCBE-BSA (4.2 mg/mL) had a characteristic peak at 275 nm. Obviously, the characteristic peak of Cd-ITCBE-BSA was significantly different from those of Cd-ITCBE and BSA. These properties indicate that Cd-ITCBE was successfully coupled to the BSA carrier protein. The result is shown in Figure 2a. Similarly, Cd-ITCBE-OVA (2.1 mg mL^−1^) was successfully synthesized. The result is shown in Figure 2b. 

### 3.2. Sodium Dodecyl Sulfate Polyacrylamide Gel Electrophoresis (SDS-PAGE) Identification of Cd-ITCBE-BSA and Cd-ITCBE-OVA

**Figure 3:**
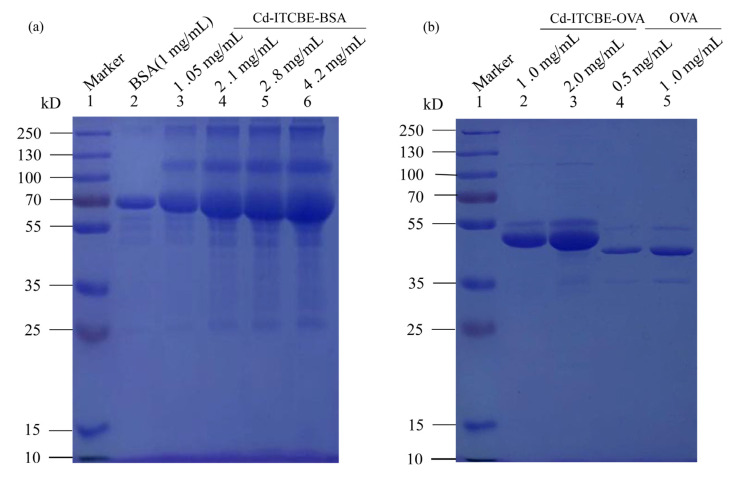
Sodium dodecyl dulfate polyacrylamide gel electrophoresis (SDS-PAGE) identification of Cd-ITCBE-BSA (**a**) and Cd-ITCBE-OVA (**b**).

The prepared Cd-ITCBE-BSA and Cd-ITCBE-OVA were identified by SDS-PAGE electrophoresis. The results showed that the Cd-ITCBE-BSA band was found after the appearance of the BSA band, reflecting a hysteresis. It showed that the relative molecular weight of Cd-ITCBE-BSA was greater than that of BSA. Based on this phenomenon, it could be inferred that Cd (II) was successfully conjugated to BSA by the bi-functional chelator ITCBE. The identification results were consistent with those obtained via UV scanning. The result is shown in Figure 3a. The Cd-ITCBE-OVA band had a significant hysteresis relative to OVA, indicating that its relative molecular weight was much greater than that of OVA. In accordance with this result, it could be concluded that Cd (II) was triumphantly coupled to OVA via the bi-functional chelator ITCBE. The result is shown in Figure 3b. 

### 3.3. Identification of Anti-Cadmium Monoclonal Antibody

Eight stable clones emerged after the fusion, and the most sensitive clones from the plate, as well as the clone 3A2, were chosen for ascite generation and further ELISA optimization. The affinity of anti-cadmium monoclonal antibody was 3.6 × 10^−5^ by ELISA.

### 3.4. Determination of Optimal Working Concentration of Monoclonal Antibody and Coated Anti-Gen for Cd (II)

The inhibition rate was calculated from the measured OD_450_ value, the blank value and the standard displayed value of 10 ng mL^−1^ Cd (II). The result is shown in Table 1. The results highlighted that the reaction values and inhibitory rates of Cd-ITCBE-BSA with different dilution concentrations were different from those of the anti-cadmium monoclonal antibody. Then, the maximum inhibition rate of the 10 ng mL^−1^ Cd (II) standard was selected within the range of OD_450_ values. Therefore, the dilution multiples of the coating anti-gens Cd-ITCBE-BSA and the anti-cadmium monoclonal antibody were 1:50,000 and 1:20,000, respectively. It was chosen as the optimal working concentration.

### 3.5. Standard Curve Linear Range and Detection Sensitivity

**Figure 4:**
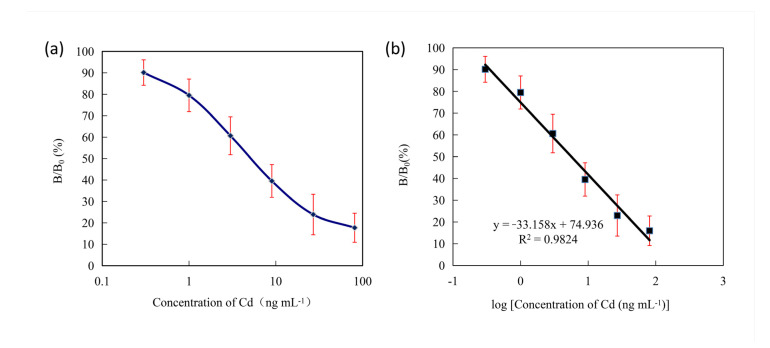
The standard representative curve (**a**) and standard calibration curve (**b**) of Cd (II) indirect competitive enzyme-linked immunosorbent assay (ic-ELISA). Each point represented the average of 12 replicates. The concentrations of Cd (II) standard solution were 0.33, 1.0, 3.0, 9.0, 27.0 and 81.0 ng mL^−1^, respectively.

The semi-logarithmic standard curve of Cd (II) ic-ELISA was established according to the experimental chromogenic numerical results. The results showed that the standard curve of the Cd (II) semi-logarithm was a typical S curve in the range of 0.33~81 ng mL^−1^. The result is shown in Figure 4a. The standard curve of linear regression was established. The result is shown in Figure 4b. The linear regression equation was *y* = −33.158*x* + 74.936 (R^2^ = 0.9824), indicating that the logarithm of the standard concentration of Cd (II) had a good correlation with the binding rate.

### 3.6. Specificity Analysis of Ic-ELISA

The cross reaction of anti-cadmium monoclonal antibody with other heavy metal ions would increase the interference of false positives. The calculation formula of the CR value was as Equation (2):

(2) CR (%) = (IC_50_ (Cd (II))/IC_50_ (compounds)) × 100%.

The result is shown in Table 2. The results showed that IC_50_ = 5.53 ± 0.76 ng mL^−1^ and IC_10_ = 0.35 ± 0.24 ng mL^−1^ based on the standard curve. The CR of the anti-cadmium monoclonal antibody compared to those of the other 12 heavy metal ions was less than 0.1%. The results showed that the anti-cadmium monoclonal antibody had little cross-reactivity with the other 12 heavy metal ions. It is clear that the ic-ELISA method established in this study has good specificity.

### 3.7. Analysis of Limits of Detection and Limits of Quantitation

Ten negative food packing material samples were detected by ICP-AES. As a control, the ic-ELISA established in this paper was used to determine Cd (II) in food packing material samples and repeated 10 times. The mean and standard deviation of 10 negative food packing material samples were calculated and analysed. The LOD and LOQ of Cd (II) residue in food packing material samples were established by ic-ELISA method. The results show that the LOD and LOQ of Cd (II) residue were 30.53 ng mL^−1^ and 35.24 ng mL^−1^, respectively. The result is shown in Table 3. Therefore, the minimum limit of quantitation of the ic-ELISA method established could meet the detection of residual Cd (II) in food packing material samples.

### 3.8. Precision and Accuracy of Ic-ELISA Analysis

The precision of ic-ELISA could be analysed by the CV values. The CV value is defined as the difference between the test material of the same stage and the test material of different stages. When the coefficient of variation was less than 10%, the experiment was stable. The CV values for each gradient concentration were calculated from 12 sets of parallel experiments, and the total mean values were calculated. The results made clear that the intra- and inter-board variation coefficients were 7.20% and 6.74%, respectively. The result is shown in Table 4. The CV values of both were less than 10%. This indicated that the ic-ELISA established in our paper had good precision.

The accuracy of ic-ELISA could be analysed by adding the recovery rates of Cd (II) standard at different concentrations in negative food packing material samples. After the addition of 0, 100, 200 and 400 ng mL^−1^ Cd (II) standards to the negative food packing material samples, the average recoveries were 92.34% ± 4.26%, 89.03% ± 10.80% and 95.81% ± 11.40%, respectively. The result is listed in Table 5. The CV values of the 10 check duplications were 4.61, 11.68 and 9.74%, respectively. On the basis of the result of our ic-ELISA method, the recovery rate and the repeat CV value were 89.03~95.81%, and 4.61%~11.68%, respectively. The apparent high accuracy can meet the requirement for the rapid detection of Cd (II) residue in food packing material samples. 

### 3.9. Comparison of Ic-ELISA and ICP-AES Detection in Spiked Sample

**Figure 5:**
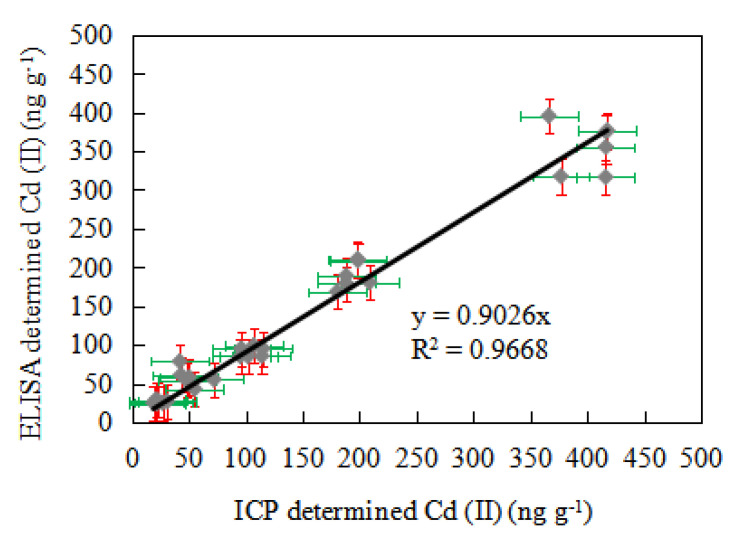
Correlation between ic-ELISA and inductively coupled plasma-atomic emission spectrometry (ICP-AES) analysis of Cd (II) in spiked food packaging samples (10 samples, with detection repeated six times).

The ICP-AES and ic-ELISA established in this paper were used to compare the difference between the two methods. The Cd (II) standards were added as experimental samples to negative food packing material samples of 50, 100, 200 and 400 ng mL^−1^, respectively. The ICP-AES and ic-ELISA tests were repeated six times in parallel. In Figure 5, the detection results of the ordinate diagram of ic-ELISA and the abscissa diagram of ICP-AES were arranged. The regression curve analysis was provided, and the regression equation was Y = 0.9026X. The correlation coefficient of the two methods was R^2^ = 0.9668. The *t*-test between the ic-ELISA and ICP-AES detections in the spiked samples was also performed, and the results show that there was no significant difference between these two groups.

### 3.10. Statistical Analysis of the 72 Actual Samples

**Figure 6:**
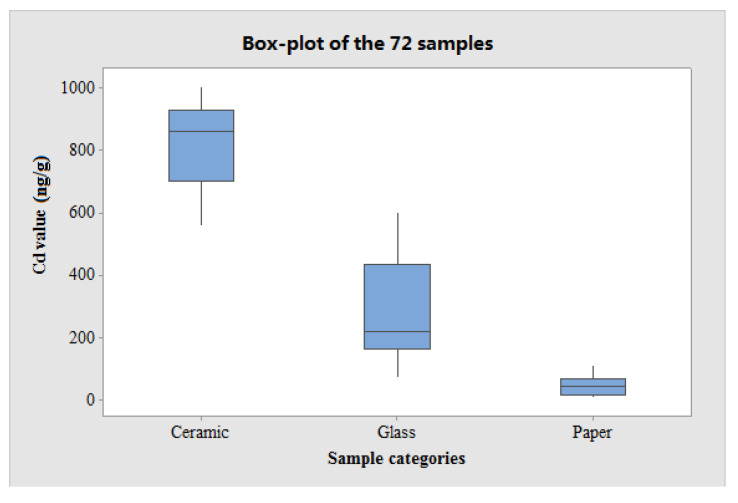
Box-plot of the 72 original samples (three categories, four samples per category and six replicates per sample).

As mentioned before, the 72 actual samples consisted of three categories of materials—ceramic, glass and paper. The mean values of Cd contained in the ceramic, glass and paper groups were 826.0 ± 133.0 ng g^−1^, 276.2 ± 164.3 ng g^−1^, and 45.5 ± 33.8 ng g^−1^, respectively. Box-plot analysis of the 72 actual samples is presented in Figure 6, where the *x*-axis represents the three categories (ceramic, glass and paper) and the *y*-axis represents the Cd concentration detected in these samples. 

As observed in Figure 6, the ceramic group has the highest Cd value, while the paper group has the lowest Cd value, which is interesting. Analysis of variance (ANOVA) was also applied, and the result demonstrated that there is a significant difference among these three groups of food packaging materials at the 0.01 level.

### 3.11. Comparison of Ic-ELISA and ICP-AES Detection in Actual Sample

**Figure 7:**
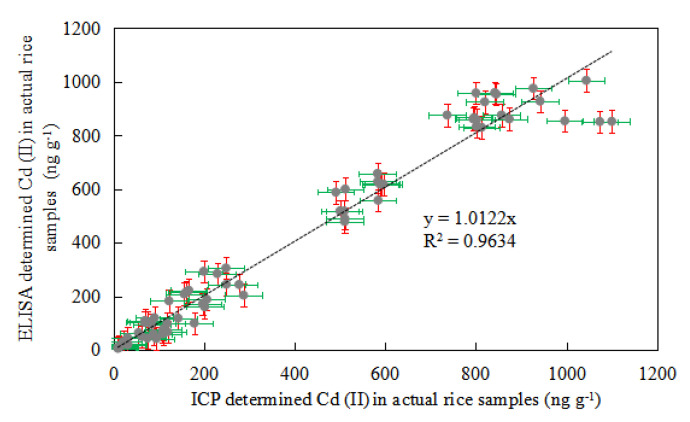
Correlation between Ic-ELISA and ICP-AES analysis of Cd (II) in actual food packaging samples (12 samples with detection repeated three times).

Detection results of the food packing samples indicated that the ic-ELISA method established could be an effective tool in the detection of actual samples. The feasibility of the ic-ELISA method was reflected by testing actual samples. Six identical samples were taken from each actual sample and repeated three times using ic-ELISA and ICP-AES. Figure 7 exhibits the test results of the ordinate graph of ELISA and the abscissa graph of ICP-AES. The regression equation was Y = 1.0122 X and the correlation coefficient of the two methods was R^2^ = 0.9634, indicating that the detection values of ic-ELISA and ICP-AES were very similar. That is to say, the detection value of ic-ELISA is close to that of the actual sample value. The *t*-test between ic-ELISA and ICP-AES detection in the actual samples was also performed, and the results show that there was no significant difference between these two groups.

## 4. Conclusions

A rapid detection method for the determination of Cd in food packing material at ng mL^−1^ levels was developed in ic-ELISA. This new approach can be completed in 40 min with simple operation. Based on the standard curve established, the UV wavelength of the enzyme marker can be quantitatively analysed. IC50 and specificity are similarly easy to calculate and analyse. The cross-reactivity with other heavy metals was low, and iC-ELISA showed better sensitivity and specificity. As shown by comparison of the results of ic-ELISA and ICP-AES, ic-ELISA can significantly improve the reliability of immunoassaying, and the precision and accuracy of Cd detection. This method provides a rapid, accurate and economical alternative tool for the detection of Cd content in food packing materials. 

## Figures and Tables

**Table 1:** The optimum concentration of anti-cadmium monoclonal antibodies and Cd-ITCBE-BSA.

Cd-ITCBE-BSA Dilution Times	Cd (II) Concentration (ng/mL)	Anti-Cadmium Monoclonal Antibody			
		1:20,000	1:40,000	1:60,000	1:80,000
1:20,000	0	2.3 ± 0.1	1.6	1.1 ± 0.1	1.0
	10	1.6 ± 0.1	0.8	0.6	0.4
	Inhibition rate (%)	31.0	46.8	43.8	60.2
1:30,000	0	2.2 ± 0.1	1.5	0.9	0.9 ± 0.1
	10	1.3 ± 0.1	0.7	0.5 ± 0.1	0.3
	Inhibition rate (%)	38.4	54.6	39.9	65.9
1:40,000	0	2.0 ± 0.1	1.3 ± 0.1	0.8	0.7
	10	0.9 ± 0.1	0.5	0.3	0.2
	Inhibition rate (%)	53.9	65.1	64.4	69.6
1:50,000	0	1.8 ± 0.1	1.2	0.8	0.7
	10	0.8 ± 0.1	0.4	0.3	0.2
	Inhibition rate (%)	56.0	67.0	66.0	73.0

**Table 2:** Cross-reactivity of anti-cadmium monoclonal antibody with Cd (II) and other metal ions.

Metal Ion	IC_50_ (ng/mL)	Cross-Reactivity (%)
Cd (II)	5.53 ± 0.76	100
Al (III)	>810	<0.1
Ca (II)	>810	<0.1
Cu (II)	>810	<0.1
Fe (III)	>810	<0.1
Hg (II)	>810	<0.1
Mg (II)	>810	<0.1
Mn (II)	>810	<0.1
Pb (II)	>810	<0.1
Zn (II)	>810	<0.1
Cr (III)	>810	<0.1
Ni (II)	>810	<0.1

**Table 3:** The limit of detection and quantification of indirect competitive ELISA in food packaging materials (*n* = 10).

Detection Item	Concentration (ng g^−1^)
Mean (X)	25.82
Standard deviation (SD)	1.57
Limit of Detection (LOD)	30.53
Limit of Quantification (LOQ)	35.24

**Table 4:** Variation of intro- and inter-assay.

Concentration of Cd (II) (ng/mL)		0	0.33	1	3	9	27	81
Intra-assay (*n* = 12)	CV (%)	2.83	5.95	8.12	4.59	9.03	11.47	8.43
	Mean	7.20%						
Inter-assay (*n* = 12)	CV (%)	4.25	6.00	7.10	7.66	9.23	6.92	5.99
	Mean	6.74%						

**Table 5:** Test of recovery of Cd (II) in food packaging materials (*n* = 10).

Spiked Concentration (ng/mL)	Average Measured Value ± SD (ng/mL)	Average Recovery ± SD (%)	CV (%)
0	25.82 ± 1.57	/	/
100	92.34 ± 4.26	92.34 ± 4.26	4.61
200	178.07 ± 21.60	89.03 ± 10.80	11.68
400	383.24 ± 45.61	95.81 ± 11.40	9.74

## Data Availability

We can provide and share experimental data in order to enable other authors to achieve best practices in archiving research data.
